# Immune Cell-Derived Extracellular Vesicles – New Strategies in Cancer Immunotherapy

**DOI:** 10.3389/fimmu.2021.771551

**Published:** 2021-12-08

**Authors:** Pengxiang Yang, Yong Peng, Yuan Feng, Zhuoying Xu, Panfeng Feng, Jie Cao, Ying Chen, Xiang Chen, Xingjian Cao, Yumin Yang, Jing Jie

**Affiliations:** ^1^ Key Laboratory of Neuroregeneration of Jiangsu and Ministry of Education, Co-Innovation Center of Neuroregeneration, Nantong University, Nantong, China; ^2^ Department of Clinical Laboratory, The First People’s Hospital of Nantong, Affiliated Hospital 2 of Nantong University, Nantong, China; ^3^ Institute of Cancer Prevention and Treatment, Heilongjiang Academy of Medical Science, Harbin Medical University, Harbin, China; ^4^ Department of Pathology, Nantong Hospital of Traditional Chinese Medicine, Affiliated Traditional Chinese Medicine Hospital of Nantong University, Nantong, China; ^5^ Department of Pharmacy, The First People’s Hospital of Nantong, Affiliated Hospital 2 of Nantong University, Nantong, China; ^6^ Department of Pathology, The First People’s Hospital of Nantong, Affiliated Hospital 2 of Nantong University, Nantong, China; ^7^ Department of Oncology, The First People’s Hospital of Nantong, Affiliated Hospital 2 of Nantong University, Nantong, China

**Keywords:** extracellular vesicles (EVs), tumor, immunotherapy, T cell, NK, macrophage, dendritic cells (DCs)

## Abstract

Immune cell-derived extracellular vesicles (EVs) have increasingly become the focus of research due to their unique characteristics and bioinspired applications. They are lipid bilayer membrane nanosized vesicles harboring a range of immune cell-derived surface receptors and effector molecules from parental cells. Immune cell-derived EVs are important mediators of intercellular communication that regulate specific mechanisms of adaptive and innate immune responses. However, the mechanisms underlying the antitumor effects of EVs are still being explored. Importantly, immune cell-derived EVs have some unique features, including accessibility, storage, ability to pass through blood-brain and blood-tumor barriers, and loading of various effector molecules. Immune cell-derived EVs have been directly applied or engineered as potent antitumor vaccines or for the diagnosis of clinical diseases. More research applications involving genetic engineering, membrane engineering, and cargo delivery strategies have improved the treatment efficacy of EVs. Immune cell-derived EV-based therapies are expected to become a separate technique or to complement immunotherapy, radiotherapy, chemotherapy and other therapeutic modalities. This review aims to provide a comprehensive overview of the characteristics and functions of immune cell-derived EVs derived from adaptive (CD4^+^ T, CD8^+^ T and B cells) and innate immune cells (macrophages, NK cells, DCs, and neutrophils) and discuss emerging therapeutic opportunities and prospects in cancer treatment.

## Introduction

Cancer is a leading cause of human death worldwide, and the vast majority of cancer patients are treated with chemotherapy and radiotherapy, which are typically only partially effective and lead to a variety of serious side effects. In contrast, the past decade has witnessed the development and validation of cancer immunotherapies that stimulate the immune system of patients to combat cancers (1, 2). The human immune system is responsible for the recognition and elimination of antigenic foreign substances and coordinates with other biological systems of the body to jointly maintain the stability of the internal environment and physiological balance (3, 4). The system is composed of immune tissues, organs, and cells and immune-active substances, which control the dynamic functions of immune surveillance, defense, and regulation. In certain types of cancer, the immune system can be both cause and cure by contributing to chronic inflammation that promotes tumor development; however, in other types of cancer, the immune system provides the ultimate weapons against metastatic disease (5, 6). Compared to conventional therapies, which directly kill both cancer and healthy cells, immunotherapy can more specifically target cancer cells *via* modulation of the functions of immune cells, causing milder side effects (7). Thus, the development of means to harness, direct, or restrain immune responses has great potential for enhancing our health and preventing future relapses (8, 9). Research focused on cancer immunology and translational immunotherapy has been bolstered by recent successes of clinical trials, including immune checkpoint antibodies, monoclonal antibodies, vaccinations, and chimeric antigen receptor (CAR)-T cell therapies (10–12).

Human immune cells belong to two functional groups: innate and adaptive cells. Innate immune cells are the first line of defense against abnormal cells, such as tumor and pathogen-infected cells (13). These innate immune cells rapidly move to the sites of infection or tissue damage and secrete potent inflammatory mediators to help destroy tumor cells (14–16). Adaptive immune cells, T and B lymphocytes complement the functions of innate immune cells. They recognize specific antigens associated with tumors and proliferate and differentiate (17, 18). Then, these cells destroy the tumor with a high degree of specificity (19). Notably, some T and B cells have long-term memory functions that prevent recurrence of tumors expressing previously encountered antigens; these cells enable protection by many vaccines for decades (20–22). Innate and adaptive responses work cooperatively to effectively clear tumors without damaging the host tissues.

The clinical success of cancer immunotherapies ultimately involves the regulation of immune cells; these treatments include tumor vaccines that modulate dendritic cells (DCs), immune checkpoint blockade therapies that enhance T cell function in the tumor microenvironment, and chimeric antigen receptor (CAR)-T cell therapies, which have been developed to stimulate tumor-specific humoral and cytotoxic T lymphocyte (CTL) responses (23–25). These treatments involve direct or indirect application of immune cells; however, many barriers to the implementation of these methods pose problems due to tumor heterogeneity and escape mechanisms. Both preclinical and clinical data revealed that DC vaccination induces effective antitumor immunity *in vivo*. However, only a limited number of patients benefit from clinical trials performed during the past two decades (15, 26). As a means of the most promising immunotherapy, CAR-T cells have also been reported to cause toxic effects, such as cytokine release syndrome, which is characterized by high fever, hypotension, hypoxia, multiorgan toxicity, and CAR-T cell-related encephalopathy syndrome (27). Systemic cell-based therapies are being studied, and multiple potential alternative approaches are being investigated.

Extracellular vesicle (EV)-based therapies have emerged as a potential option for current cancer due to their pathophysiological efficacy. The ongoing clinical trials of cancer immunotherapy based on EVs are listed in **Table 1** (28). EVs are nanometric membrane vesicles that are secreted by cells in the body, including almost all immune cells. EVs have some unique functions, including accessibility, storage, passing through the blood-brain and blood-tumor barriers, loading various effector molecules, and combining with other therapeutic modalities (29, 30). Multiple studies have examined tumor cell-derived vesicles as important mediators of intercellular communication that regulate specific mechanisms of tumor survival, growth, angiogenesis, and metastasis (31). Immune cell-derived EVs carry a range of functional molecules, and various EV-based strategies are being developed for applications in preclinical studies, including genetic engineering, membrane engineering, and cargo delivery (32, 33). Thus, immune cell-derived EV treatment is a separate or complementary technique for immune cell-based therapy. The present review is specifically focused on the structural features and major effects of innate and adaptive immune cell-derived EVs. The roles of these EVs in mediating immune regulation provide new ideas for the future diagnosis and treatment of cancers.

**Table 1:** The part of ongoing clinical trials of cancer immunotherapy based on EV.

ID	Sponsor	Tumor	Enrollment	Strategy	Phase/Status
**Immunotherapy**					
NCT01159288	Gustave Roussy, Cancer Campus, Grand Paris	NSCLC	41	Cyclophosphamide and tumor antigen-loaded Dex	Phase 2/Complete
NCT03608631	M.D. Anderson Cancer Center	Pancreas cancer	28	EVs With KrasG12D siRNA	Phase 1/Recruiting
NCT01550523	Jefferson University	Recurrent malignant gliomas	13	EVs deliver tumor antigens, activate immune response	Phase 1/Complete
**Diagnosis**					
NCT03824275	Columbia University	Prostate cancer	300	Diagnostic marker	Phase 2/3/Recruiting
NCT03228277	Konkuk University Medical Center	NSCLC	25	Marker after treatment	Phase 2/Complete
NCT02977468	Eileen Connolly	TNBC	15	Marker after treatment	Phase 1/Recruiting
**Drug delivery**					
NCT01294072	University of Louisville	Colon cancer	35	Plant EVs Deliver Curcumin	Phase 1/Recruiting

The data source: https://clinicaltrials.gov/. NSCLC, Non small cell lung cancer; TNBC, Three-negative breast cancer.

## Biological Characteristics of Immune Cell-Derived Vesicles

Immune cell-derived vesicles are heterogeneous in size, originate from cells, and are detected in the blood, urine, saliva, and cerebrospinal fluid (34). Based on their biogenesis mechanism, EVs are classified into three types: exosomes, microvesicles and apoptotic bodies. These three types of vesicles are different in diameter; microvesicles are generally larger in size with a diameter of approximately 100 nm to 1 μm, and exosomes have a diameter of 30-150 nm. Apoptotic bodies derived from apoptotic cells have a diameter of 1-5 μm EVs (30, 35, 36). A summarized list of the characteristics of each vesicle is provided in **Table 2** (37–39). In this review, we focus on exosomes and microvesicles that come from immune cells and refer to them as EVs in general.

**Table 2:** Major types of extracellular particles.

Vesicle	Size (nm)	Origin	Markers
Exosomes	30-150	Endosomes	Tetraspanins, Alix, TSG101, CD63
Microvesicles	100-1000	Plasma membrane	Integrins, selectins, CD40
Apoptotic bodies	1000-5000	Plasma membrane, endoplasmic reticulum	Phosphatidylserine, genomic DNA, receptors

## Isolation and Identification

It was critical to obtain a large number of EVs with high purity and quickly to meet the demands of basic research and clinical application. The most commonly used method is ultracentrifugation, based on EV density. Initially, large dead cells and cell debris were eliminated. Then, the supernatant was centrifuged for 70 min at 10^5^ × g to pellet EVs. The final pellet was washed in PBS to eliminate contaminating proteins. In addition, immune-affinity antibody capture techniques, size-dependent gradient centrifugation, ultrafiltration and precipitation were also used to isolate EVs from diverse organic samples. A list of characteristics of each isolation method is summarized in **Table 3** (29, 40, 41).

**Table 3:** Isolation methods of EVs.

Isolation Methods	Purity	Principle	Characters
Ultracentrifugation	High	Density	Large acquisition
Density-gradient centrifugation	High	Density	Cost time
Immune-affinity capture	High	Biomarker	High cost
Ultrafiltration	Moderate	Size	Easy and fast
Precipitation	Low	Precipitation	Contaminants

The routine methods of EV identification include western blotting and flow cytometry. Several markers are commonly used for immunoblot analysis, such as tetraspanins (CD9, CD63, and CD81), a protein involved in multivesicular biogenesis (Tsg101), and a cytoskeleton-associated protein (ezrin). Moreover, EVs can be characterized by physical and morphological characteristics, including scanning electron microscopy (SEM), transmission electron microscopy (TEM), cryoelectron microscopy (cryo-EM), dynamic light scattering (DLS), atomic force microscopy (AFM), resisting pulse sensing (RPS) and nanoparticle tracer analysis (NTA) (42–44).

## Innate Immune Cell-Derived EVs

The antitumor response requires the participation of innate and adaptive immune cells. Innate immune cells include monocytes/macrophages, neutrophils, natural killer (NK) cells, NKT cells, γδT cells, eosinophils, basophils, and mast cells (45, 46). DCs are professional antigen-presenting cells known to play a key role in the initiation and maintenance of antitumor immunity, bridging innate and adaptive immune responses (47). The functions of EVs derived from NK cells, macrophages, DCs and neutrophils are summarized in detail separately.

### Natural Killer Cell-Derived EVs

NK cells are innate immune effector cells that play an important role in human organ immunosurveillance, cancer, or pathogen infections (48). NK cells express germline-encoded activating and inhibitory surface receptors that tune NK cell-mediated cytotoxicity by sensing changes in the extracellular microenvironment (35, 48). Under steady-state conditions, NK cell activity is stringently controlled by membrane-expressed inhibitory receptors binding to human leukocyte antigen (HLA) molecules, which block activating receptors of NK cells from binding to specific molecules (49). These receptors form activating immunological synapses with target cells through surface receptors, including NKp46, NKp30, NKp44, NKG2D, and DNAM-1, and utilize their cytotoxic ability to eliminate abnormal cells at an early stage of tumorigenesis or infection. When tumor cells or viruses dominate the local microenvironment at a late stage, inhibitory receptors, such as KIRs and NKG2A/CD94, represent an important mechanism that limits the cytotoxic effects of autologous NK cells (29, 50, 51).

**Figure 1:**
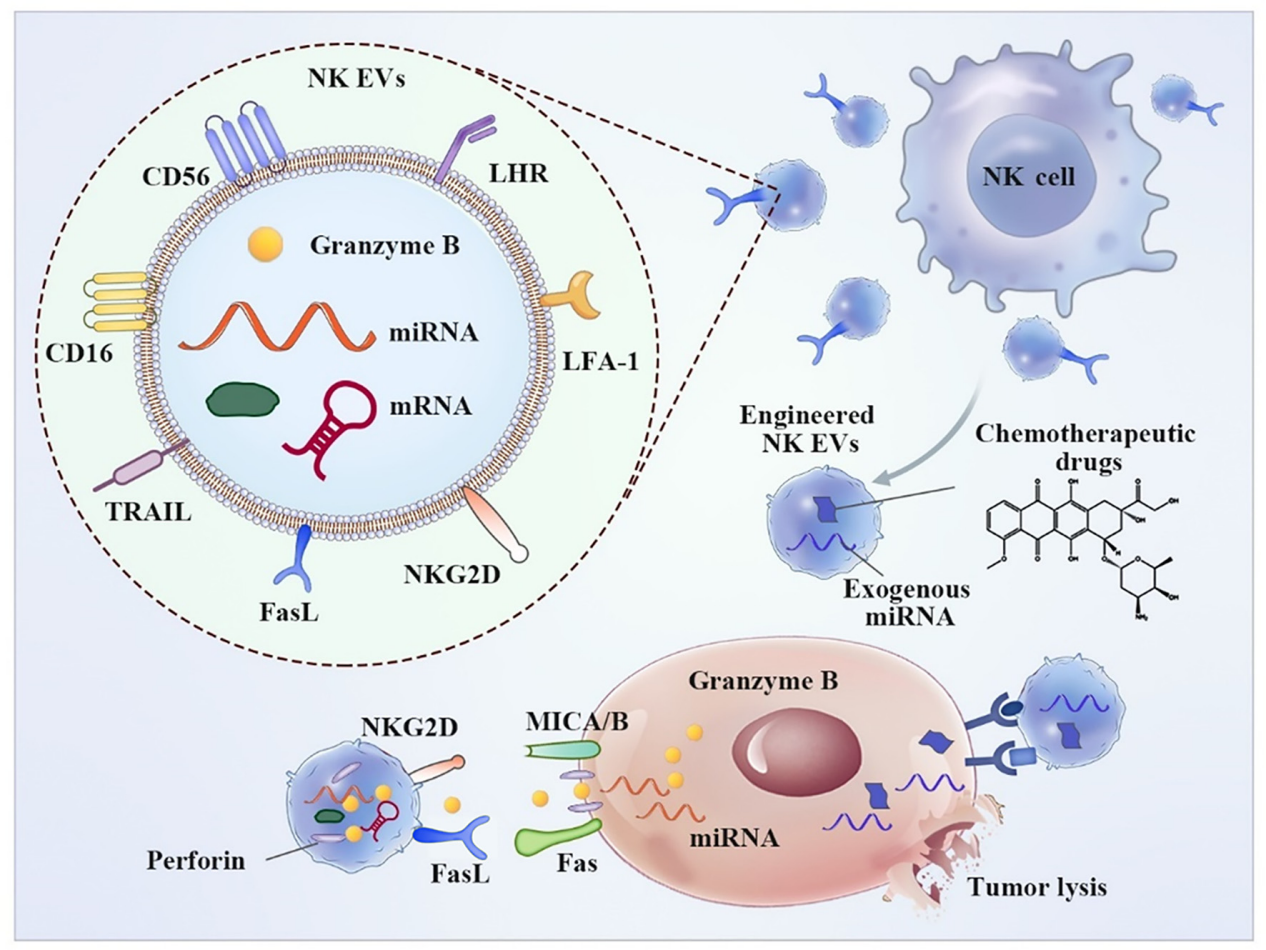
Typical characteristics and anti-tumor application of NK-derived EVs. NK EVs binds tumor cells through NKG2D-MICA/B and exhibit cytotoxic effect resulting from a cargo of released cytotoxic proteins, including perforin, granzymes and small antimicrobial peptides, resulting tumor cell apoptosis. Additionally, Engineered NK EVs-coated nanoparticle was employed for chemotherapeutic drug delivery.

NK-derived EVs contain typical NK surface receptors that perform a function similar to that of parental cells (**Figure 1**). The release of active NK EVs is able to induce apoptosis of tumor cells. In contrast, NK ligand-bearing tumor cells induce downregulation of the expression of active receptors, such as NKG2D, and inhibit degranulation on NK cells, resulting in compromised cytotoxicity and reduced levels of antitumor immune surveillance and lytic proteins (52). EVs released from NK cells deliver a cargo of cytotoxic proteins, including perforin, granzymes, granulysin, FasL/CD178, TNF-related apoptosis-inducing ligand (TRAIL/CD253) and small antimicrobial peptides (53). These effector molecules destroy target cells, including breast cancer, melanoma, and hematologic malignancies, *via* a well-known mechanism of the direct killing pathway (54, 55).

NK-derived EVs contain other molecules involved in cellular homing, adhesion, and immune activation that cause indirect tumor killing. Immunomodulation studies revealed that NK-derived EVs mainly function by stimulating peripheral blood mononuclear cells (PBMCs) and increasing the fraction of CD56^+^ NK cells (48). A study by Paolo Neviani and coworkers showed that NK-derived EVs carrying the tumor suppressor miRNA-186 are cytotoxic against neuroblastoma cell lines. Targeted delivery of miRNA-186 directly inhibits the expression of oncogenes, including MYCN, AURKA, TGFBR1, and TGFBR2, and prevents TGFβ1-dependent immune escape in high-risk neuroblastoma patients (56). Yoon-Tae Kang et al. reported the fabrication of a novel microfluidic system based on an NK-graphene oxide chip. The chip combined patient-specific NK cells and biogenesis of NK-derived EVs. NK-derived EVs exhibited cytotoxic effects on circulating tumor cells (CTCs). This versatile system is expected to be used for patient-specific NK-based immunotherapies against CTCs for potential prognostic/diagnostic applications (57).

### Macrophage-Derived EVs

**Figure 2:**
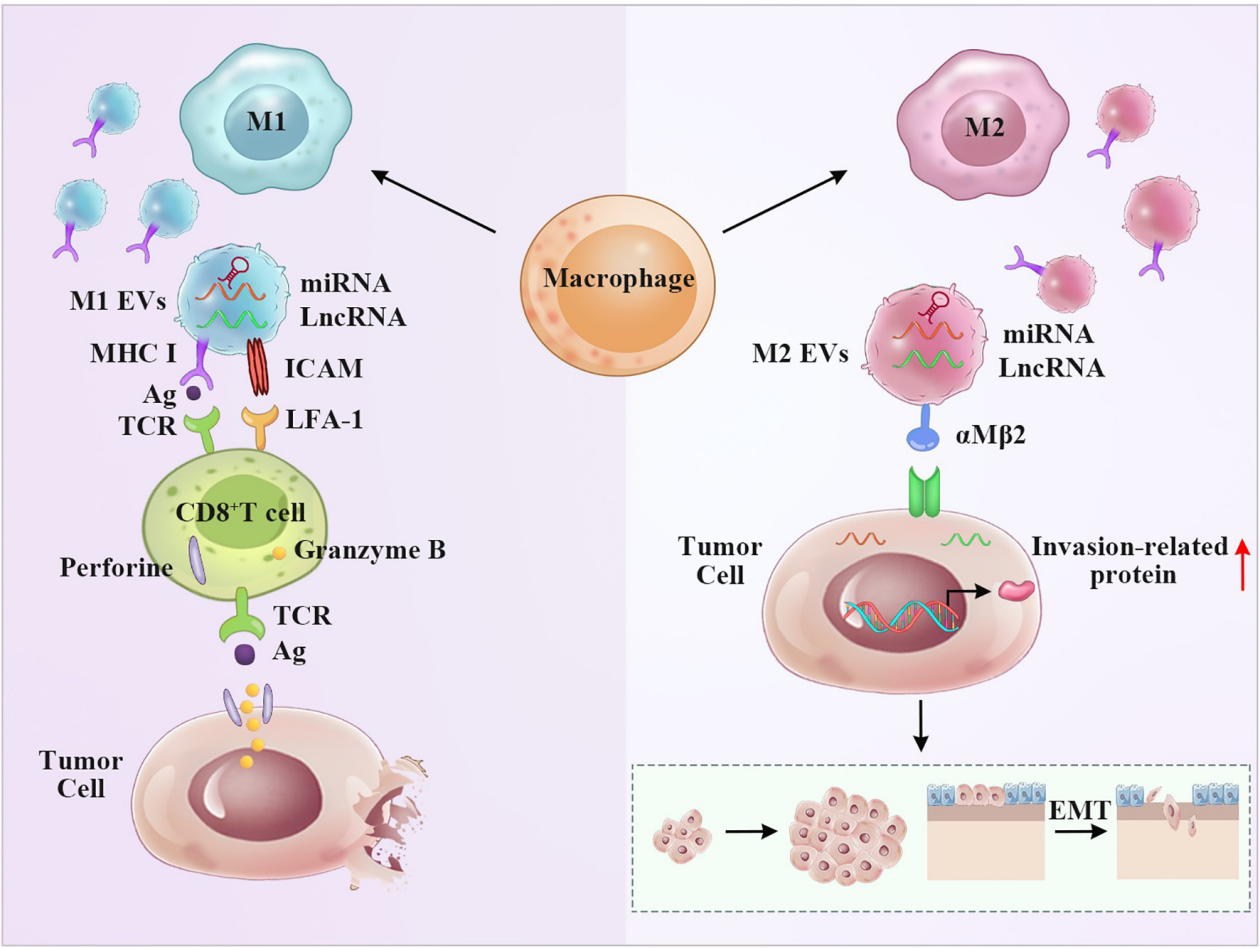
M1 and M2 macrophage-derived EVs display the opposite anti-tumor effect. Left panel: The presence of MHC and ICAM molecules on the surface of M1 EVs give them the potential to stimulate T cells, resulting T cell activation and tumor apoptosis. The miRNA and LncRNA derived from M1 EVs may aid this process. Right panel: M2 EVs transferred miRNA and LncRNA to regulate invasion-related protein, thus promoting the invasion and metastasis of tumor.

Macrophages express various functional programs in response to various microenvironmental signals. As multifunctional cells, macrophages infiltrate tumor tissues (tumor-associated macrophages, TAMs) and play an important role in tumor initiation and progression. Proinflammatory M1 macrophages and “alternatively activated” anti-inflammatory M2 macrophages represent the extremes of a continuum of functional states (58, 59). Clinical and experimental evidence has shown that M1 macrophages phagocytose tumor cells and that M2 macrophages promote tumor growth and metastasis (60). Macrophages absorb antigens that are released by EVs and subsequently deliver them to CD4^+^ or CD8^+^ T cells. Receptor cells bind EVs due to receptor-ligand interactions (61). Certain surface ligands and adhesion molecules, such as tetraspanins, ICAM-1, and phosphatidylserine, are involved in the process (**Figure 2**). Macrophage-derived EVs have multiple functions depending on various phenotypes of parental cells. Both endogenous and exogenous stimulatory factors influence the secretion of macrophage-derived EVs (62). Lysosomes can fuse with multivesicular bodies to determine their trafficking pathway. Therefore, vesicles highly depend on the functions of lysosomes (63). Other factors, such as autophagy and aging, can also influence the contents of macrophage-derived EVs (64, 65). The hypoxic microenvironment is a common feature of solid tumors and can augment the release of macrophage-derived EVs (61).

Macrophage-derived vesicle-mediated cell-to-cell interactions can mediate the exchange of miRNAs, long noncoding RNAs (lncRNAs), and proteins. Noura Ismail and coworkers demonstrated that miRNA-223 contained in macrophage-derived EVs was transported to the target cells and induced the differentiation of macrophages (66). Zhengtian Li et al. demonstrated that miRNA-16-5p derived from M1 macrophage-derived EVs enhanced the T cell-dependent immune response by decreasing the expression of PD-L1, which inhibited gastric cancer formation *in vitro* and *in vivo* (67). MiRNA-12-5p and miRNA-155-5p were present at high levels in M2 macrophage-derived EVs, which were transferred to colorectal cancer cells; these EVs bound to the cells and decreased the expression of BRG1, leading to cancer cell migration and invasion, as demonstrated by Jingqin Lan et al. (68).

LncRNAs in macrophage EVs modulate the tumor microenvironment and participate in tumor pathogenesis. For example, Lei Wu et al. demonstrated that M2 macrophage-derived EVs carried the lncRNA-PVT1 sponge miRNA-21-5p to upregulate SOCS5, which alleviated inflammation and protected EAE mice by repressing the JAK/STAT3 pathway (69). Xifeng Mi et al. demonstrated that M2 macrophage-derived EVs carried the lncRNA AFAP1-AS1 sponge miRNA-26a to upregulate ATF2, promoting the invasion and metastasis of esophageal cancer (70). Thus, these results provide a new point of view in which macrophage-derived EVs carrying lncRNAs participate in tumor pathogenesis. Macrophage-derived EVs are also packaged with a variety of protein effector molecules, such as ERAP1 and CCL3, to enhance phagocytic functions. TNF-α and IFN-γ are crucial for nitric oxide (NO) synthesis, which also facilitates vesicle-mediated macrophage functions (71). Integrin αMβ2 contained in M2 macrophage-derived EVs is notably specific and efficient and contributes to the migration of hepatocellular carcinoma by activating the MMP-9 signaling pathway (**Figure 2**) (72). Importantly, vesicle-mimetic nanovesicles derived from M1 macrophages can repolarize M2 macrophages to M1 macrophages. Enhancement of the antitumor efficacy of aPD-L1 and suppression of tumor growth result from the release of proinflammatory cytokines (73).

### Dendritic Cell-Derived EVs

DCs are the most powerful antigen-presenting cells in the human body that can activate resting T cells, building an essential bridge between innate and adaptive responses (14, 74, 75). Tumor-proximal DCs can capture the antigens generated and released during tumorigenesis and present captured tumor-associated antigens (TAAs) in cooperation with costimulatory molecules, such as CD80 and CD86, through the major histocompatibility complex (MHC)-I and MHC-II molecules to naïve CD8^+^ T cytotoxic cells and naïve CD4^+^ T helper cells, respectively, leading to the initiation and activation of antitumor immune responses (76, 77).

**Figure 3:**
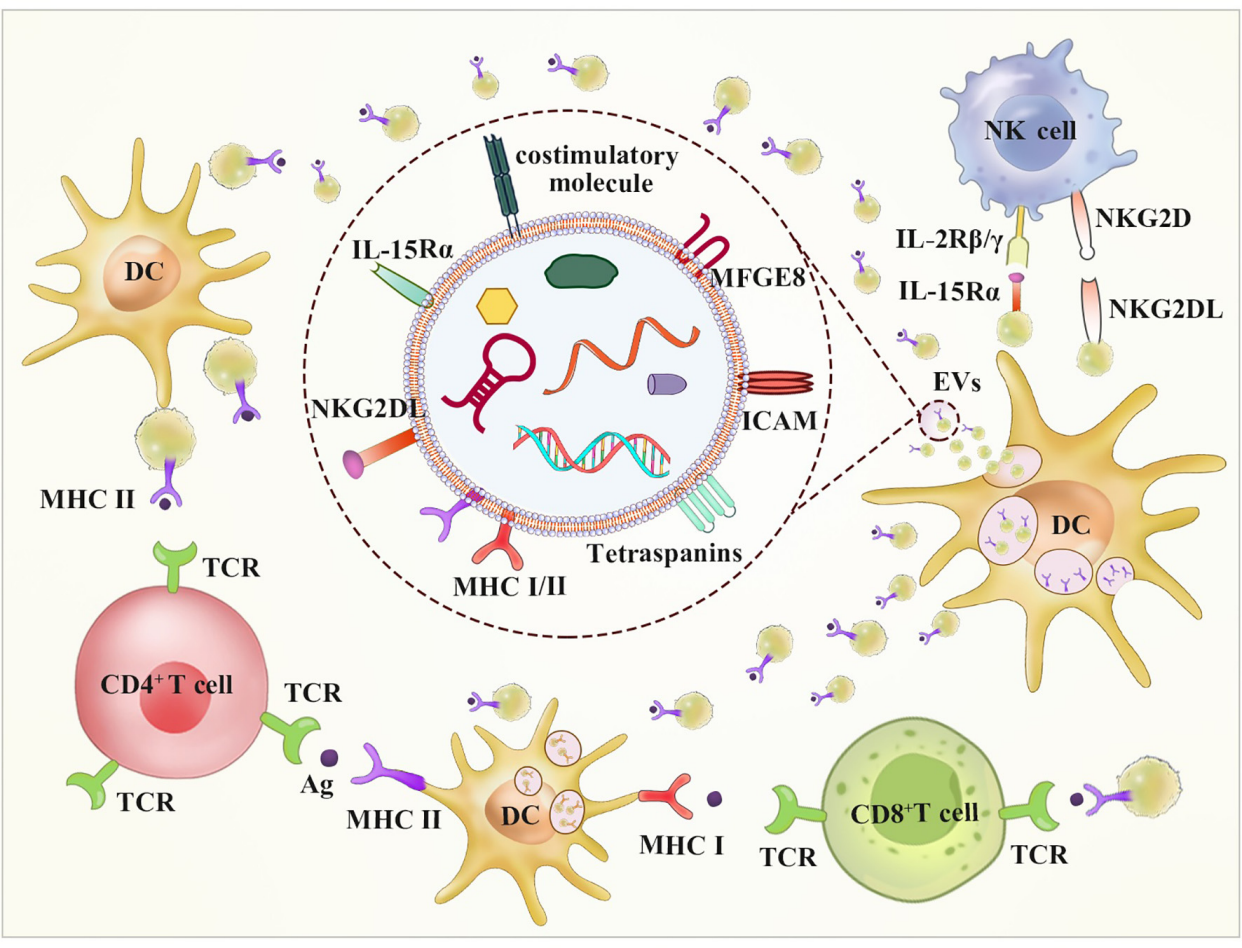
DC-derived EVs may stimulate both CD8^+^ and CD4^+^ T cells by direct and indirect routes. A route for DC EVs stimulation of T cells occurs directly *via* the expression of MHC-I, MHC-II and costimulatory molecules on the surface of it. The indirect way stimulation of T cells occurs *via* bystander DCs through two mechanisms. The first way involves EVs internalization and transfer of antigen-MHC complex. The other way called cross-dressing involves antigen-MHC complex direct transfer to DC surface. Additionally, DC EVs have been shown to possess NKG2D-L and the IL-15/IL-15Rα complex, which can result in NK activation.

DC-derived EVs are small lipid vesicles that have been used to stimulate antitumor immune responses in mouse models and clinical trials. Näslund showed that protein-loaded DC-derived EVs activated CD8^+^ T cell and B cell responses *in vivo* antitumor immunity (78). A phase II clinical trial involving the administration of tumor antigen-loaded Dex in NSCLC has been completed (28). EVs contain the CD1a, b, c, and d proteins, which are involved in cross-presentation of lipid antigens (40, 79). Importantly, the tumor antigen peptide-MHC complex and costimulatory factors, such as CD86, are expressed at high levels on the surface of mature DC-derived EVs, which can be presented to immune cells to activate TAA-specific effector T cells (80, 81). EVs contain a variety of membrane proteins, such as αMβ5, milk fat globule-EGF factor 8 protein (MFGE8), and intercellular cell adhesion molecule-1 (ICAM-1), which can be targeted to bind and fuse to immune cells (DCs, T cells, and NK cells) with high levels of integrin αvβ5 receptor expression (82). Additionally, the ligand of the NK cell activating receptor (NKG2D) is expressed at high levels on the surface of DC-derived EVs, which can directly activate NK cells *in vitro* and *in vivo* in a non-MHC-dependent manner to exert an antitumor effect (83). DC-derived EVs also express Toll-like receptors (TLR1/2 and TLR4) on their surface, which enhance the expression of transmembrane tumor necrosis factor and activation of bystander DCs, leading to the production of proinflammatory cytokines and subsequent activation of NK cells (**Figure 3**) (84).

DC-derived EVs contain heat shock proteins (HSPs) that are involved in antigen presentation. HSC73, a member of the HSP70 family, together with HSP90, is present at a high abundance in the cytosolic fraction of DC-derived EVs and can bind antigens to load onto MHCs (85). Additionally, DC-derived EVs contain various metabolic enzymes, such as lipid kinases, peroxidases, enolase-1, and pyruvate (86). In addition to proteins, DC-derived EVs contain various RNAs, which facilitate intercellular communication and induce posttranslational modifications. Importantly, miRNAs delivered by EVs are functional because they suppress targeted mRNAs in acceptor DCs (87).

In addition to proteins, DC-derived EVs mediate cell-to-cell interactions and exchange miRNAs. Various miRNAs can be exchanged depending on the maturation of DCs. Angela Montecalvo et al. demonstrated that 63 miRNAs are differentially expressed in immature versus mature DC-derived EVs (87). Qingshan Ji et al. demonstrated that miRNA-21 delivered by vesicles derived from thymic stromal lymphopoietin-treated DCs regulates Th17/Treg differentiation by inhibiting smad7 (88). Zhongliu Cao et al. reported that miRNA-335 delivered by vesicles derived from mature DCs enhances the proliferation and osteogenic differentiation of marrow-derived mesenchymal stem cells by targeting LATS1, and this effect was accompanied by inhibition of Hippo signaling (89). Engineering DC-derived EVs expressing miRNAs, which modulate signaling pathways, may enhance antitumor activity (40, 90). Moreover, EVs have distinct advantages over DC-based therapy and have highly promising prospects for immunotherapy.

Many factors influence the production and release of DC-derived EVs, including the maturation stage of DCs, stimulatory signals produced by T cells, and DNA-damaging treatment. C Théry and coworkers reported that the production of EVs downregulated DC maturation (85). Sophie Viaud et al. demonstrated that MHCI, MHCII, and costimulatory factors are more abundant in EVs derived from mature DCs (91). Stimulatory signals produced by T cells encountering immature DCs may trigger a transient increase in EV secretion (92). However, the DNA-damaging signal *via* TSAP-6 regulates protein secretion, leading to a severe compromise of the production of DC-derived EVs (82). The phenotype and immunogenicity of EVs are critical for their function. IFN-γ, IL-3, and IL-4 are used for DC maturation, whereas GM-CSF/IL-4 and GM-CSF/IL-10 inhibit DC maturation. EVs maintain the same phenotype as parental DCs, leading to antitumor effects or to inhibition of inflammation.

Previous studies demonstrated that DC-derived EVs can initiate potent antitumor immunity *via* direct or indirect pathways (**Figure 3**). DC-derived EVs directly present tumor antigen-MHC complexes to T cells with low efficiency (93). EVs merge the DC surface membrane and deliver the tumor antigen peptide-MHC complex, which is called a cross-dressing process, to be recognized by T cells without the need for antigen uptake. This approach results in a stronger antitumor effect than that achieved by direct presentation (82). Moreover, DC-derived EVs can deliver some tumor antigens in the form of proteins or long peptide chains directly to DCs, which perform antiuptake, processing, and presentation (93). Furthermore, Graziela Gorete Romagnoli et al. reported that DC-derived EVs can turn tumor cells into immunogenic targets to deliver immune function-associated molecules to cancer cells, resulting in extensive proliferation of previously sensitized IFN-γ-secreting T cells (94).

DC-derived EVs have attracted attention in cancer immunotherapy because they activate both T and B cells to induce antitumor immunity *in vivo*. CD8^+^ T cells are extensively activated by the EV TAA-MHCI complex (78). Various strategies, such as chemical adjuvants, IFN-γ, and αGC, which boost DC maturation, can remarkably promote an increase in IFN γ-producing CD8^+^ T cells and enhance the level of IL-2 (95). CD4^+^ T cell propagation was extensively initiated by the vesicle TAA-MHCII complex through an indirect pathway when DCs were loaded with a protein rather than a peptide antigen. Ben J C Quah and coworkers demonstrated that primary B cells can also propagate upon stimulation of EVs derived from mycoplasma-infected DCs, and these effects do not involve CD40, LPS, or the CpG signaling pathway (96).

### Neutrophils-Derived EVs

According to the spatiotemporal production mechanism, EVs derived from neutrophils can be divided into two subtypes, neutrophil-derived trails (NDTRs) and neutrophil-derived microvesicles (NDMVs). NDTRs are produced by migrating neutrophils, while NDMVs are produced by neutrophils that have migrated to the site of inflammation (97). Further studies have found that the neutrophil production of NDTRs and NDMVs depends on features of the immune environment, such as interactions between adhesion molecules, rather than on the type of stimulation (98, 99). The two types of EVs have similar characteristics, including surface markers, stimulating factors and bactericidal activity (100). Both types of EVs kill bacteria through ROS- and granule-dependent mechanisms (97). However, integrin-mediated interactions are necessary for the production of NDTRs, and the production of NDMVs mainly depends on the PI3K pathway. Although NDTRs and NDMVs share the most common markers, studies have found that NDMVs express CD16 at relatively high level, while NDTRs express PSGL-1 and Fcγ type III receptor at relatively high levels (101). Although both types of EVs are easily taken up by monocytes, NDTRs induce the polarization of M0 macrophages toward a proinflammatory phenotype, while NDMVs induce their polarization toward an anti-inflammatory phenotype (100, 102). Differential expression analysis of miRNAs in NDTRs and NDMVs revealed that NDTRs contain proinflammatory miRNAs, such as miR-4454, miR-1260, miR-7975 and miR-1285, whereas NDMVs contain anti-inflammatory miRNAs, such as miR-451a, miR-150 and miRNA-126. This result indicates that neutrophils may integrate different types of miRNAs into EVs according to the immune environment (100). In addition, neutrophil-derived EVs such as granules can have a certain killing effect and provide defense against invading pathogens. Moreover, neutrophil-derived EVs have a short life span and can be easily handled, making them very advantageous for use as drug carriers.

## Adaptive Immune Cell-Derived EVs

The adaptive immune response mainly involves T lymphocyte-mediated cellular responses and B lymphocyte-mediated humoral immunity (103). These lymphocytes play a major role in the antitumor immune response. The functions of EVs derived from T and B immune cells are summarized in detail separately.

### CD4^+^ T Cell-Derived EVs

T lymphocytes are immune cells that play critical roles in carrying out and bolstering the immune response against pathogens, the self, allergens, and cancers (104). T cells can be classified into various subsets according to their immune phenotype, mainly CD4^+^ T helper cells and cytotoxic CD8^+^ T cells. CD4^+^ T cells can be further divided into Th1, Th2, Th9, Th17, Th22, follicular helper T cells (Tfhs), and regulatory T cells (Tregs), and each of these groups produces specific effector cytokines under unique transcriptional regulation (105).

The tetraspanin family of proteins, such as CD63, CD9, and CD81, are mainly used as EV markers on the membranes of T cells. Moreover, the membranes of T cells contain many function-related molecules, including CD2, CD3/TCR, CD4, CD8, CD11c, CD25, CD69, LFA-1, CXCR4, FASL, and GITR (106). These membrane proteins are involved in the activation, proliferation, differentiation, antigen presentation, and effector functions of the cells. T cell-derived EVs unidirectionally transfer miRNA from T cells to antigen-presenting cells (107). Furthermore, activated T cell-derived EVs are delivered to DNA-primed DCs through antigen-driven contacts (108). CD4^+^ T cell-released EVs potentiate the efficacy of the hBsAg vaccine by enhancing B cell responses (109).

Regulatory Treg-derived EVs have received widespread attention due to their ability to exert immunosuppressive effects, as they were shown to prolong the survival of a kidney allograft rat model (110). Okoye et al. found that Tregs could suppress effector T cells by delivering miRNAs. Treg-derived EVs contain premature and mature miRNAs, particularly with proapoptotic or antiproliferative functions (31). Isobel S Okoye al. reported that the microRNA Let-7d was preferentially packaged into Treg EVs and transferred to Th1 cells, thereby suppressing Th1 cell proliferation and IFN-γ secretion (111). In addition to microRNAs, regulatory Treg-derived EVs contain CD25, CTLA-4, and CD73. CD73-positive Treg EVs were shown to convert extracellular denosine-5-monophosphate to adenosine. Once adenosine binds to its receptors on activated effector T cells, it suppresses cytokine production and T cell responses (41). Therefore, regulatory Treg-derived EVs have potential as a target for cancer immunotherapies.

### CD8^+^ T Cell-Derived EVs

The functions of CD8^+^ T cell-derived EVs depend on their parental cell subpopulations and activation status. Fully activated CTLs enhance the activation of low-affinity CTLs through EV secretion in immunotherapy for cancers and chronic viral infections (112, 113). Moreover, Yufan Qiu et al. recently reported that activated T cell-derived exosomal PD-1 attenuates PD-L1-induced immune dysfunction in TNBC, providing a potential therapeutic strategy to attenuate the suppressive tumor immune microenvironment (114). However, Xiaochen Wang et al. demonstrated that functionally exhausted CD8^+^ T cells could secrete vast EVs, which can be taken up by normal CD8^+^ T cells, and impaired their proliferation (Ki67), cell activity (CD69) and the production of cytokines such as interferon-γ and interleukin-2. Microarray detection identified 257 candidate lncRNAs derived from exhausted CD8^+^ T cells, which regulate diverse processes related to CD8^+^ T cell activity, such as metabolism, gene expression, and biosynthesis (115).

**Figure 4:**
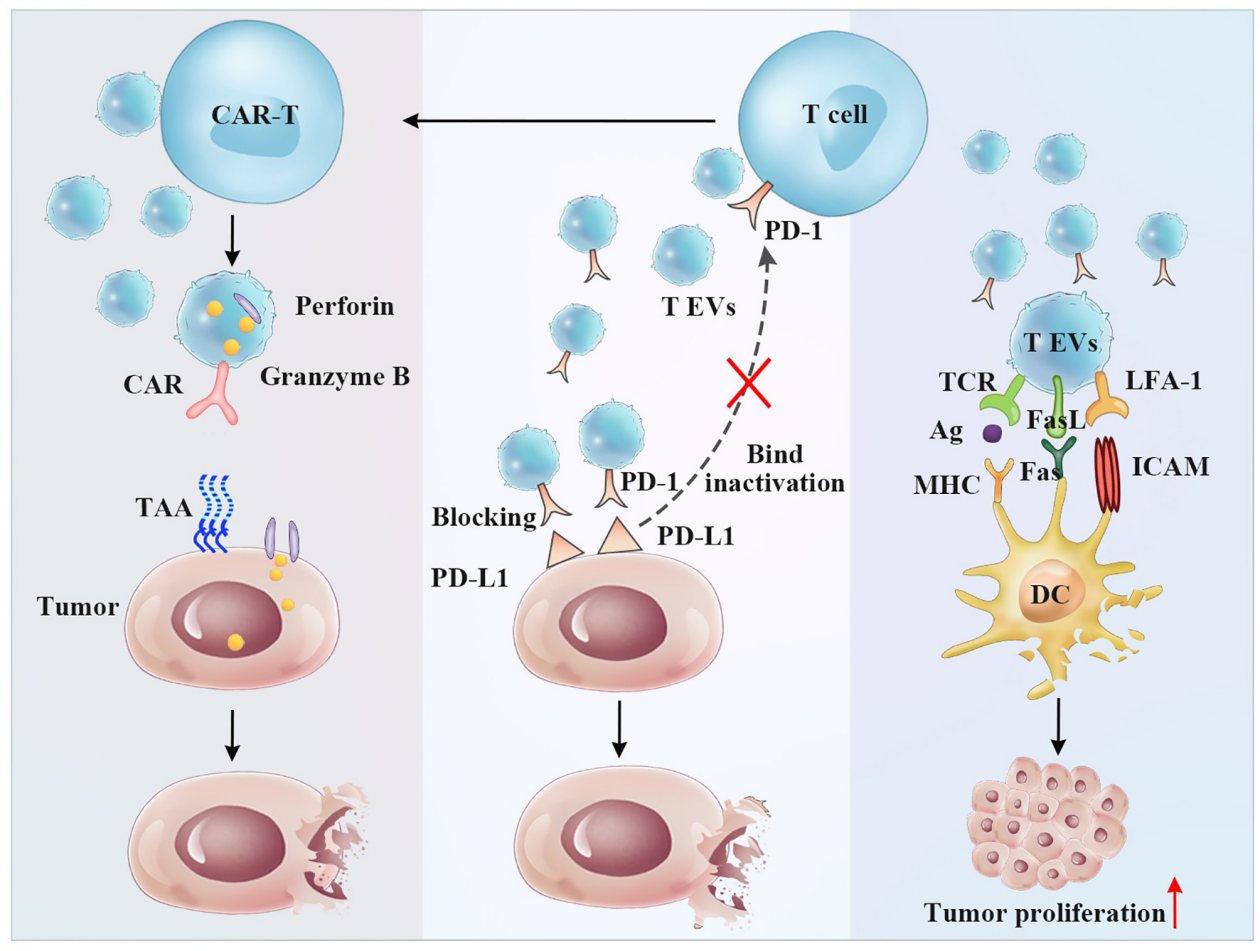
The antitumor and immunoregulatory effects of T cell-derived EVs. Left panel: CAR-T cell EVs induce antitumor effect by CAR-targeted tumor cells and secreting perforin and granzyme B Middle panel: T EVs exposing the PD-1 receptor can bind PD-L1 expressing tumor cells preventing T cell inactivation. Right panel: T EVs exposing FasL receptor can bind Fas expressing DCs resulting DCs apoptosis and tumor proliferation.

However, in many cases, CD8^+^ T cell subtype-derived vesicles show higher immunosuppressive properties in tumors, which is inconsistent with the functions of the corresponding source cells. EVs from activated CD8^+^ T cells were shown to activate ERK and NF-κB in melanoma cells, leading to increased MMP9 expression and promoting cancer cell invasion *in vitro*, suggesting a role for T cell-derived vesicles in tumor progression (116). In addition, Hua Min et al. reported that EVs derived from irradiated esophageal carcinoma-infiltrating T cells promote the metastasis of esophageal cancer cells by inducing the epithelial to mesenchymal transition (117). All these studies documented that T cell-derived vesicles may play an important role in tumor formation and invasion. However, it is well known that the functions of T cell-derived EVs may be influenced by an unfavorable tumor microenvironment. Studies on the immunological enhancement of EVs are essential for cancer treatment (**Figure 4**).

One of the approaches involves the separation of single T cells by cell sorting to obtain T cell subsets with high viability and purity. Wen-Jie Zhou et al. reported that CD45RO^-^CD8^+^ T cell-derived EVs release more miRNA-765 than CD45RO^+^CD8^+^ T cells. In therapeutic studies, these vesicles limit estrogen-driven disease development *via* regulation of the miRNA-765/PLP2 axis (118). Additionally, T cell-derived EVs carry the effector molecules perforin and granzyme. Selective targeting and therapeutic properties of anticancer agents will be of great benefit due to genetic engineering. Currently, CAR-T cells have been widely recognized by the medical community as a novel form of viable tumor treatment due to their high efficacy in cancer therapy. However, as a means of systemic cell-based therapy, CAR-T cells have been reported to induce toxic effects, such as cytokine release syndrome, which is characterized by high fever, hypotension, hypoxia, and multiorgan toxicity (119, 120). A recent study showed that CAR-T-derived vesicles (CAR vesicles) can be used for cancer immunotherapy because they express CAR and cytokine molecules that evoke significant antitumor effects (**Figure 4**). CAR vesicles were also shown to cause fewer side effects, such as cytokine release syndrome, and to lack functional suppression by PD-L1 (44). These vesicles may have several other advantages, including accessibility, storage, passing through physical barriers, and an ability to be combined with traditional treatments (43). CAR-T cell-derived EVs, as a cell-free treatment, which is a complementary technique for immune cell-based therapy, have a lower risk of toxicity than living CAR-T cells.

### B Cell-Derived EVs

In addition to antibody production, B cells also play roles in antigen presentation and in the activation and regulation of T cells and innate immune effector cells. B cells can secrete antigen-presenting vesicles under the stimulation of CD40, CD40L, interleukins, interferons, and tumor necrosis factor, among others. Among many factors, the TCR-MHCII interaction plays a major role in the release of EVs from B cells (121, 122). In many cases, B cell-derived EVs provide immunogenic stimulation. Raposo discovered that B cells can secrete antigen-presenting vesicles, and some molecules, such as MHCII, B7, LFA-3, and ICAM, are expressed on the vesicle membrane to facilitate CD4^+^ T cell activation (123). B cell-derived EVs are also involved in CTL activation. Sarah C used DH LMP2A mice to demonstrate that the BCR plays an important role in the induction of effective CTL responses by B cell vesicles (124). However, Matthew W showed that a human B cell-derived lymphoblast-like cell line (LCL) expresses MHCII^+^ FasL^+^ EVs at very high levels, which can induce CD4^+^ T cell apoptosis (123). Zhang et al. found that CD19^+^ EVs from B cells contain high levels of CD39 and CD73, which hydrolyze the ATP released by tumor cells after chemotherapy into adenosine and attenuate the effect of chemotherapy by inhibiting the CD8^+^ T cell response (125). The above evidence shows that the antitumor role of B cell-derived EVs is complicated. Follicle dendritic cells (FDCs) in lymphoid follicles are another potential target of B cell-derived EVs. MHCII, FcR, and integrin α4β1, which play important roles in the germinal center B cell-FDC interaction, are highly enriched in B cell EVs (126).

## Engineering Immune Cell-Derived EVs

Novel insights into the biological functions of immune cell-derived EVs has paved the way for the efficient production of engineered EVs as potent antitumor vaccines and for specific functional applications. Engineered technologies include genetic engineering, membrane engineering and cargo delivery strategies. These methods are applied to the parent cell to promote their secretion of genetically modified EVs or directly to the EVs themselves. Many studies have used tumor-derived EVs; however, little is known about whether these exosomes have potential negative effects (127). Immune cell-derived EVs have improved safety and functionality profiles and serve as an emerging therapeutic strategy for cancer treatment.

### Engineering of EVs Content

Due to the small size of EVs, many investigators have engineered donor cells and then isolated the EVs containing miRNAs, antigens, cytokines or drugs of interest (128). MiRNAs have various biological functions and play important roles in tumor immunotherapy. Functional miRNAs are overexpressed in parental cells to enhance the load of secreted EVs through nonviral or viral methods. EVs ensure that the content remains intact and lessen degradation upon transfer to recipient cells in miRNA and anti-miRNA therapies (127). O’ Brien et al. found that miRNA-134 was downregulated in breast tumors and played a role in controlling Hsp90. miRNA-134 was then overexpressed in the cell, and the secreted EVs were then isolated. Exosomes enriched with miR-134 reduced the invasion and migration of breast cancer cells and enhanced their sensitivity to anti-Hsp90 drugs (129).

Recently, an increasing number of studies have focused on vesicles as drug delivery carriers. Engineered NK-derived vesicles may be used to support tumor therapy (**Figure 1**). D Han et al. used NK-derived EV-entrapped paclitaxel to enhance the antitumor effect of the drug (130). Guosheng Wang et al. reported a “cocktail therapy” strategy based on excess NK-derived EVs in combination with biomimetic core-shell nanoparticles for tumor-targeted therapy. The nanoparticles were self-assembled and had a dendrimer core loaded with a therapeutic miRNA and a hydrophilic EV shell (131). Engineered macrophage vesicle-coated nanoparticles were also employed for drug delivery in triple-negative breast cancer (TNBC) treatment targeting the epithelial to mesenchymal transformation factor c-Met, which was overexpressed in TNBC, resulting in significantly improved efficiency of cellular uptake and inhibition of tumor growth (132). Sagar Rayamajhi and coworkers constructed a macrophage-derived vesicle-mimetic hybrid for the delivery of doxorubicin for breast cancer treatment. Hybrid vesicles, with sizes less than 200 nm, can deliver drugs in acidic cancer environments and demonstrate prominent toxicity against breast cancer cells (133). These results indicated that engineered vesicles will be a promising drug delivery platform for tumor treatment.

### Engineering of EVs Surface

As mentioned in the preceding text, immune cell-derived EVs express the majority of surface receptors on their parental cells. These signaling molecules on the membrane surface help EVs find ligand molecules of target cells and release their load. For instance, proteins such as CD80, CD86, and ICAM1, which are involved in T cell costimulation, also accumulate in DC-derived EVs (29, 134). Macrophage-secreted EVs can transfer their surface antigens to DCs, thereby promoting the activation of CD4^+^ T cells (135). Inspired by this, EV surface modification has also been employed. The engineered EV surface displays a special functional peptide or glycolipid fragment, which accumulates in tumors or lesion organs through active targeting.

Genetic engineering is also a reliable and commonly used method. Y Tian et al. modified immature DC-derived EV surfaces by introducing the pEGFP-C1-RVG-Lamp2b plasmid, which fused the iRGD peptide (CRGDKGPDC) to the N-terminus of the murine membrane protein Lamp2b. The engineered iRGD peptide exhibited a highly efficient targeting ability and delivered doxorubicin to breast cancer cells, resulting in the inhibition of tumor growth (136). We previously generated human CAR constructs encoding an MSLN-targeted and Myc-tagged scFv. The second-generation CARs were designed with a transmembrane region and signaling domain and were transduced *via* a lentiviral vector. The genetically engineered T cell-derived EVs maintained most of the characteristics of their parental T cells, including the surface expression of CAR. CAR-carrying EVs inhibited the growth of MSLN-positive triple-negative breast cancer (TNBC) cells, and no obvious side effects were observed (43). These results suggest that EVs that allow proper membrane protein function are promising options for clinical treatment.

## Tumor-Derived EVs on Immune Cells

Numerous immune cell types, including T/B cells and DCs, emerge in tumor-infiltrating tissues (3, 7). Tumor-derived EVs affect the functions of immune cells. The contents and membrane composition of tumor-derived EVs are also similar to those of parental cells expressing tumor-specific antigens and immunostimulatory and immunosuppressive signaling molecules, thus have both antitumor and protumor effects (41). Tumor-derived EVs, as tumor antigens, are taken up by MHC I molecules on antigen-presenting cells and presented to T cells to activate antitumor responses. A typical example is the use of glioma-derived EVs to induce DC maturation and immunization in mice and thereby induce specific CD8^+^ T cell antitumor effects (137). In addition, EVs derived from HSP70-positive tumors stimulate TNF-α production in macrophages, leading to the migration and cytolytic activity of NK cells and macrophages (41). Although evidence suggests that tumor-derived EVs have antitumor effects, and opposing point of view does exist. Immunosuppressive signaling molecules on the surface of tumor cells, such as PD-L1, bind to PD-1 on the surface of activated T cells to induce the apoptosis of activated antitumor T cells, thereby facilitating tumor escape from immune surveillance (138). Douglas D et al. found that EVs shed from ovarian tumors express FasL, leading to the loss of T cell CD3-ζ expression and T cell fas-dependent apoptosis (139). Tumor-derived EVs expressing the NKG2D ligand downregulate the expression of NKG2D, weaken the cytotoxic effects of NK cells and CD8^+^ T cells, and promote tumor invasion and metastasis (140). Tumor-derived EVs also block the maturation of DCs and macrophages through a TGF-β1-dependent mechanism and promote the proliferation of Treg cells (41, 141).

## Conclusion and Perspectives

Cancer immunotherapy has emerged as a promising alternative to conventional therapies to treat a variety of malignancies and has demonstrated remarkable clinical results. Immune cell-derived EVs are gaining considerable attention as potential cancer treatment candidates (142). At present, numerous studies have focused on EVs, and their structure, formation, secretion, and functions have uncovered a significant role of EVs as intercellular communication messengers (36, 39). The other aspects of EV functions are poorly understood due to unclear mechanisms. However, EVs derived from immune cells have been successfully used to treat solid and nonsolid tumors in laboratory and preclinical studies (143).

EVs are an ideal tool for diagnostic and therapeutic markers. Many studies have shown abnormal levels of EVs in the body fluids of patients with cancer or other diseases, including blood, urine, ascites, and saliva (51). EVs can easily travel through the bloodstream due to the composition of their membrane and nanosize effects. Unique markers, such as specific RNAs and proteins, from their parental cells can be identified after EVs are isolated. Immune cell-derived EVs are also used for immune diagnosis (48, 144). Circulating immune cell-derived EVs can be disease-specific biomarkers of inflammation and tumorigenesis. The levels of these EVs in the sera are correlated with the severity of chronic hepatitis, fatty liver, etc. In the case of therapeutic interventions, the ability of immune cell-derived EVs to kill tumors is unstable and depends on the state and concentration of the extract (145). Various strategies are used to improve the treatment and reduce the side effects. (1) In genetic engineering strategies, IL-4, FasL, or IDO can be genetically transferred into DCs, and overexpressing DC-derived EVs are able to target specific tumors. (2) Membrane engineering strategies involve meticulous regulation of the membrane phospholipid composition or insertion of a targeting antibody on the surface of the EV membrane. This process can be accomplished *via* chemical crosslinking using various ligand/receptor molecules. (3) Cargo delivery strategies involve miRNAs, siRNAs, chemotherapeutic drugs, or antigens loaded into EVs. Immune cell-derived EVs are novel promising vaccines or adjuvant candidates for the treatment of cancer (42, 143). DC-derived EVs loaded with an antigen or adjuvant can induce specific CD4^+^ and CD8^+^ T cell reactions as vaccines. A combination of immune cell-derived EVs, such as NK cells and CTLs, and antitumor drugs was shown to inhibit proliferation and migration. More work is required to understand the complex functions of immune cell-derived EVs in the tumor and disease microenvironments. Nonetheless, continued breakthroughs will allow immune cell-derived EVs to emerge as novel cancer treatments to benefit patients.

## Author Contributions

PY, YP, YF, and ZX were in charge of research and drafting. PF, JC, YC, and XC helped in revision. XJC, YY, and JJ were responsible for leading this work and revising the manuscript. All authors contributed to the article and approved the submitted version.

## Funding

This work was supported by the National Natural Science Foundation of China (No. 31900987), the Heilongjiang Natural Science Foundation (No. YQ2019H022), Research Project of Health Commission of Nantong (No. MB2021011), Jiangsu Province “Double Innovation Plan” (No. JSSCBS20211603), the Nantong Science and Technology Plan Project (No. JC2019146), the Nantong University Clinical Medicine Project (No. 2019JZ004). Jiangsu Pharmaceutical Association-HengRui Hospital Pharmacy Fund (No. H202047), and the Nantong Pharmaceutical Association-Changzhou Fourth Medicine Pharmacy Research Fund (No. ntyx2020).

## Conflict of Interest

The authors declare that the research was conducted in the absence of any commercial or financial relationships that could be construed as a potential conflict of interest.

## Publisher’s Note

All claims expressed in this article are solely those of the authors and do not necessarily represent those of their affiliated organizations, or those of the publisher, the editors and the reviewers. Any product that may be evaluated in this article, or claim that may be made by its manufacturer, is not guaranteed or endorsed by the publisher.
